# Genome-wide profiling of 5-hydroxymethylcytosines in circulating cell-free DNA reveals population-specific pathways in the development of multiple myeloma

**DOI:** 10.1186/s13045-022-01327-y

**Published:** 2022-08-16

**Authors:** Brian C.-H. Chiu, Zhou Zhang, Benjamin A. Derman, Jason Karpus, Liangzhi Luo, Sheng Zhang, Spencer S. Langerman, Madina Sukhanova, Parveen Bhatti, Andrzej Jakubowiak, Chuan He, Wei Zhang

**Affiliations:** 1 Department of Public Health Sciences, The University of Chicago, Chicago, IL 60637 USA; 2 Department of Preventive Medicine, Northwestern University Feinberg School of Medicine, Chicago, IL 60611 USA; 3 Section of Hematology/Oncology, Department of Medicine, The University of Chicago, Chicago, IL 60637 USA; 4 Department of Chemistry, The University of Chicago, Chicago, IL 60637 USA; 5 Department of Pathology, Northwestern University Feinberg School of Medicine, Chicago, IL USA; 6 Department of Cancer Control Research, BC Cancer Research Institute, Vancouver, BC V5Z1L3 0611 Canada; 7 Department of Biochemistry and Molecular Biology, Institute for Biophysical Dynamics, and Howard Hughes Medical Institute, The University of Chicago, Chicago, IL 60637 USA

**Keywords:** Multiple myeloma, 5-hydroxymethylcytosine, Racial disparity, Epigenetic modification

## Abstract

Multiple myeloma (MM) and its precursors monoclonal gammopathy of undetermined significance (MGUS) and smoldering myeloma (SMM) are 2–3 times more common in African Americans (AA) than European Americans (EA). Although epigenetic changes are well recognized in the context of myeloma cell biology, the contribution of 5-hydroxymethylcytosines (5hmC) to racial disparities in MM is unknown. Using the 5hmC-Seal and next-generation sequencing, we profiled genome-wide 5hmC in circulating cell-free DNA (cfDNA) from 342 newly diagnosed patients with MM (*n* = 294), SMM (*n* = 18), and MGUS (*n* = 30). We compared differential 5hmC modifications between MM and its precursors among 227 EA and 115 AA patients. The captured 5hmC modifications in cfDNA were found to be enriched in B-cell and T-cell-derived histone modifications marking enhancers. Of the top 500 gene bodies with differential 5hmC levels between MM and SMM/MGUS, the majority (94.8%) were distinct between EA and AA and enriched with population-specific pathways, including amino acid metabolism in AA and mainly cancer-related signaling pathways in EA. These findings improved our understanding of the epigenetic contribution to racial disparities in MM and suggest epigenetic pathways that could be exploited as novel preventive strategies in high-risk populations.

**Supplementary Information:**

The online version contains supplementary material available at 10.1186/s13045-022-01327-y.

To the editor,

Multiple myeloma (MM) typically progresses from the precursor conditions of monoclonal gammopathy of undetermined significance (MGUS) and smoldering myeloma (SMM). Compared with European Americans (EA), African Americans (AA) are 2–3 times more likely to be diagnosed with MM. Genetic susceptibility, socioeconomic factors, and obesity do not fully explain the excess risk in AA. Clinical variations of MM between EA and AA suggest a biological cause of racial/ethnic disparities [1]. Although the importance of epigenetics to MM is recognized, previous studies have not investigated genome-wide 5-hydroxymethylcytosines (5hmC), a cytosine modification with a distinct genomic distribution and regulatory role from the more-investigated 5-methylcytosines (5mC) [2]. Reduced global 5hmC levels have been found in MM [3] and MM-specific hydroxymethylome is associated with cell proliferation and prognosis [4]. To improve understanding of the role of 5hmC in disparities in MM, we conducted a genome-wide 5hmC profiling using the 5hmC-Seal and the next-generation sequencing in circulating cell-free DNA (cfDNA) samples from 227 EA and 115 AA patients with newly diagnosed MM, SMM, and MGUS prospectively enrolled at the University of Chicago Medical Center between 2010 and 2017. (Additional file 1: Table S1; Additional file 2).

Overall, the captured 5hmC modifications in cfDNA were more abundant in gene bodies and depleted at the promoter regions (Fig. 1A). Using the Roadmap Epigenomics Project annotations as reference, we found that patient-derived 5hmC profiles were enriched in B-cell and T-cell-derived enhancer marks: H3K4me1 and H3K27ac (Fig. 1B).

**Fig. 1:**
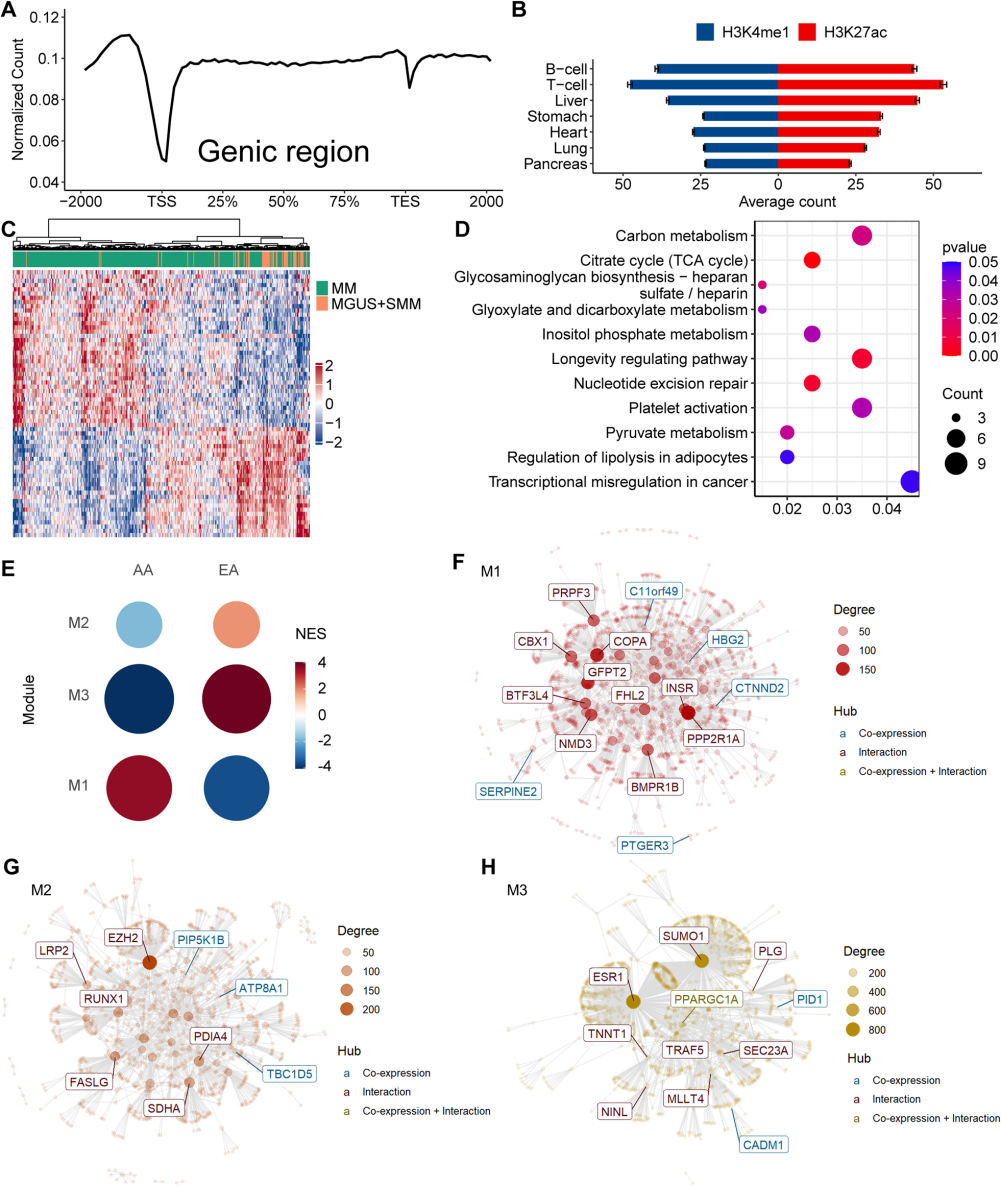
Genome-wide profiling of 5hmC from cfDNA derived from EA and AA patients with MM and its precursors. Genome-wide 5hmC was profiled in patient-derived plasma cfDNA samples using the 5hmC-Seal and the next-generation sequencing. The 5hmC-Seal data summarized for gene bodies were the primary targets for differential analysis between MM and its precursors (MGUS + SMM, i.e., MGUS and SMM combined) in all samples, using multivariable logistic regression models, controlling for age, sex, and self-reported race/population. In addition, we performed differential analysis between EA and AA patients with MM only. **A** The captured 5hmC-Seal reads in cfDNA are more abundant in gene bodies relative to the flanking regions and depleted at the promoter regions, based on the GENCODE annotations (hg19). TSS: transcription start site; TES: transcription end site. **B** The captured 5hmC-Seal reads are enriched in histone modifications marking enhancers (H3K4me1 and H3K27ac) derived from B-cells and T-cells compared with other tissue types. The annotations for H3K4me1 and H3K27ac were obtained from the Roadmap Epigenomics Project. The standard error is shown as the error bar. **C** The heat map shows the top 63 differential gene bodies between MM and its precursors in the combined EA and AA patients. **D** Shown are the enriched KEGG pathways among the top 500 differential gene bodies between MM and its precursors in the combined EA and AA samples. The X-axis represents the ratio between the number of differential genes and the total genes in a given pathway. **E** The Co-expression Network Enrichment Analysis was performed for differential gene bodies between EA and AA to provide further biological insights. Specifically, three modules (Module 1: 254 genes; Module 2: 156 genes; and Module 3: 75 genes) are shown from the modular gene co-expression analysis using the top 500 differential gene bodies between AA and EA patients with MM as the input. NES: normalized enrichment score. **F–H** Shown are the protein–protein interaction networks constructed for the co-expression and/or interaction modules identified from differential gene bodies between EA and AA patients with MM

Comparing MM and its precursors (MGUS + SMM), we identified 63 differential gene bodies at 5% FDR (false discovery rate) (Fig. 1C; Additional file 3: Table S2) after controlling for sex, age, and race/ethnicity. The KEGG pathway analysis identified several metabolism-related pathways (e.g., citrate cycle) that have been implicated in myeloma cell growth and proliferation as well as the pathogenesis of MM (Fig. 1D) (Additional file 3: Table S3).

Next, we identified 259 differential gene bodies (5% FDR) between EA and AA patients with MM (Additional file 4: Fig. S1; Additional file: 3: Table S4), of which 183 showed higher 5hmC modification levels in AA patients. Of note, *LIN28A* is one of the most frequently mutated genes reported in MM [5], while *KANSL1*, *LRRC37A3*, and *ARL17B* are in a region (chr17q21) with a segmental duplication that is primarily found in European descents [6]. We identified several metabolism related KEGG pathways (Additional file 3: Table S5). The co-expression network analysis revealed three modules showing different direction of enrichment between AA and EA (Fig. 1E). Furthermore, the protein–protein interaction network analysis identified several relevant hub genes (Fig. 1F–H). For example, *FHL2* (Module 1) has been found to regulate hematopoietic stem cell functions [7] and the production of IL6 [8], a cytokine critical to myeloma cell proliferation. Low expression of FHL2 has also been associated with development of IgM myeloma [9].

We then compared MM and its precursors in EA and AA patients separately. We identified 36 and 4 differential gene bodies (5% FDR) in EA and AA patients, respectively (Fig. 2A, B; Additional file 3: Table S6-7). Simulation analyses showed that the number of shared differential genes between EA and AA reached a peak around the 500th rank (Fig. 2C). The majority (94.8%) of the top 500 differential gene bodies were distinct between EA and AA (Fig. 2D), suggesting that although there is mechanistic commonality of myelomagenesis, racial/ethnic heterogeneity exists. The pathway analysis of the top 500 differential gene bodies showed population-specific KEGG pathways (Fig. 2E; Additional file 3: Table S8), including various cancer-related signaling pathways in EA patients, but primarily metabolism-related pathways in AA patients. For example, *PIK3CA*, which was enriched in several cancer-related signaling pathways in EA patients only, is important for constitutive Akt activity in MM cells and the blockade of PIK3CA induces cell death [10]. In contrast, several *ALDH* family genes were enriched in metabolism-related pathways in AA patients only. Increased expression of *ALDH1* in MM has been identified as a marker of tumor-initiating cells and is associated with chromosomal instability [11]. Specific transcriptional networks related to metabolisms have also been found to contribute to plasma cell growth and proliferation [12].

**Fig. 2:**
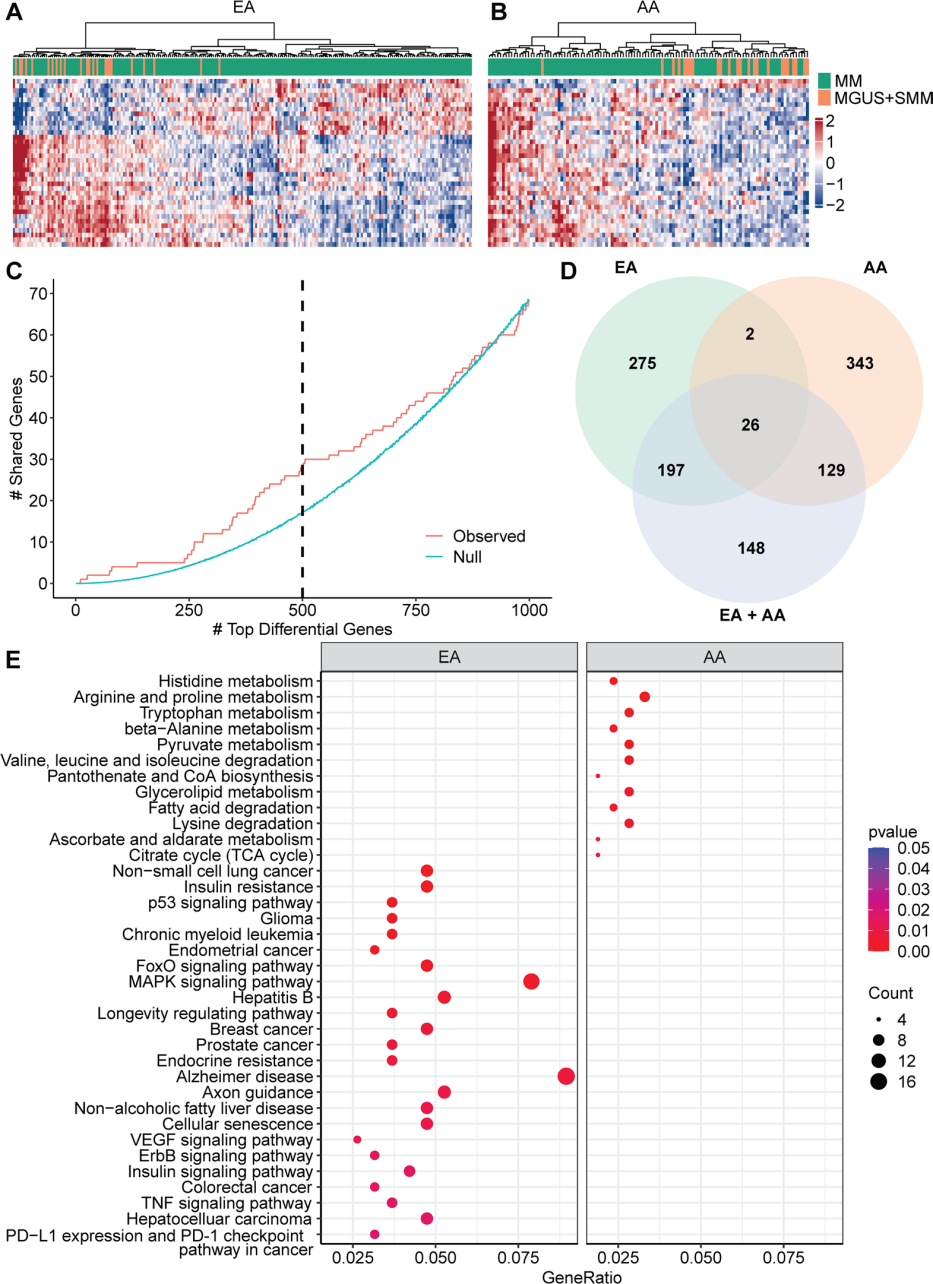
Differential analysis reflects population-specific 5hmC signatures and pathways between MM and its precursors. The 5hmC-Seal profiles summarized for gene bodies were compared between MM and its precursors (MGUS + SMM, i.e., MGUS and SMM combined) in AA and EA samples, separately, using multivariable logistic regression models, controlling for age and sex. **A** The heat map shows the top 36 differential genes between MM and its precursors in EA patients. **B** The heat map shows the top 36 differential genes between MM and its precursors in AA patients. **C** Shown is the number of shared differential gene bodies between MM and its precursors in individual populations (AA vs. EA), compared with the null distribution. The blue line represents the mean of a null distribution generated by permutation (*N* = 10,000). The red line represents the observed number of shared differential gene bodies. **D** The Venn Diagram shows the number of shared gene bodies (top 500) between MM and its precursors in EA, AA, and the combined samples. **E** Shown are the enriched population-specific KEGG pathways for the top 500 differential gene bodies between MM and its precursors in EA and AA

In conclusion, we identified population-specific 5hmC signatures and pathways that improved our understanding of the epigenetic mechanisms underlying the disparities in MM. These findings could be exploited for novel preventive strategies in high-risk populations in the future.

## Supplementary Information

**Additional file 1: Table S1**. Characteristics of study subjects, UChicago Multiple Myeloma Epidemiology Study, 2010-2017.

**Additional file 2**. Materials and Methods.

**Additional file 3:**. **Table S2.** Top 500 differentially modified gene bodies between MM and its precursors in the combined samples. **Table S3.** Enriched KEGG pathways among differentially modified genes between MM and its precursors in the combined samples. **Table S4.** Top 500 differentially modified gene bodies between AA and EA patients with MM. **Table S5.** Enriched KEGG pathways among differentially modified genes between EAs and AAs with MM. **Table S6.** Top 500 differentially modified gene bodies between MM and its precursors in EA patients. **Table S7.** Top 500 differentially modified gene bodies between MM and its precursors in AA patients. **Table S8.** Enriched KEGG pathways among differentially modified genes between MM and its precursors in different populations.

**Additional file 4: Fig. S1**. Supplementary results for the differential analysis between AA and EA patients with MM. Differential analysis was performed between AA and EA patients with MM for each gene body (5hmC modification levels, i.e., the normalized 5hmC-Seal read counts), using multivariable logistic regression models, controlling for age and sex. The heat map shows the 259 differential gene bodies at 5% FDR between AA and EA patients with MM.

## Data Availability

The raw and processed 5hmc-Seal data in the current study are available from the corresponding authors on reasonable request.
