# Supplementary material for: Genome-wide profiling of 5-hydroxymethylcytosines in circulating cell-free DNA reveals population-specific pathways in the development of multiple myeloma
Source: J Hematol Oncol. 2022 Aug 16;15:106. doi: 10.1186/s13045-022-01327-y (PMC9380317; doi:10.1186/s13045-022-01327-y)
Supplement: Supplementary file 1 — Additional file 1: Table S1. Characteristics of study subjects, UChicago Multiple Myeloma Epidemiology Study, 2010-2017. [file 13045_2022_1327_MOESM1_ESM.docx]

**Table S1.** Characteristics of study subjects, UChicago Multiple Myeloma Epidemiology Study, 2010-2017.

|  | **MGUS^+^** | | | **SMM^+^** | | | **MM^+^** | | |
| --- | --- | --- | --- | --- | --- | --- | --- | --- | --- |
|  | **Whites**  **(n=11)** | **Blacks**  **(n=19)** | ***p*-value^++^** | **Whites**  **(n=13)** | **Blacks**  **(n=5)** | ***p*-value** | **Whites**  **(n=203)** | **Blacks**  **(n=91)** | ***p*-value** |
| **Age, yrs**  Mean ± 1 SD | 68.2 ± 7.6 | 65.5 ± 8.7 |  | 65 ± 11 | 63 ± 17 |  | 61.0 ± 9.3 | 62.3 ± 10.4 |  |
|  |  |  | 0.38 |  |  | 0.85 |  |  | 0.29 |
| **Gender, Males** | 4 (36%) | 5 (26%) | 0.56 | 1 (7.7%) | 2 (40%) | 0.099 | 115 (57%) | 32 (35%) | <0.001 |
| **No. High-risk cytogenetics*** |  |  | 0.044 |  |  | 0.63 |  |  | 0.20 |
| 0 | 7 (63.6%) | 4 (21.1%) |  | 6 (46%) | 3 (60%) |  | 99 (48.8%) | 56 (61.5%) |  |
| 1 | 1 (9.1%) | 1 (5.3%) |  | 2 (15%) | 0 (0%) |  | 32 (15.8%) | 11 (12.1%) |  |
| 2+ | 0 (0%) | 0 (0%) |  | 0 (0%) | 0 (0%) |  | 21 (10.3%) | 9 (9.9%) |  |
| Unavailable | 3 (27.3%) | 14 (73.7%) |  | 5 (38%) | 2 (40%) |  | 51 (25.1%) | 15 (16.5%) |  |
| **R-ISS^^^** |  |  | 0.14 |  |  | 0.97 |  |  | 0.36 |
| 1 | 2 (18.2%) | 0 (0%) |  | 3 (23%) | 1 (20%) |  | 35 (17.2%) | 18 (19.8%) |  |
| 2 | 4 (36.4%) | 4 (21.1%) |  | 3 (23%) | 1 (20%) |  | 75 (36.9%) | 41 (45.1%) |  |
| 3 | 0 (0%) | 1 (5.3%) |  | 0 (0%) | 0 (0%) |  | 5 (2.5%) | 1 (1.1%) |  |
| Missing | 5 (45.5%) | 14 (73.7%) |  | 7 (54%) | 3 (60%) |  | 88 (43.3%) | 31 (34.1%) |  |
| **Estimated GFR^&^, (mL/min/1.73 m^2^)**  Mean ± 1 SD | 48 ± 27 | 51 ± 32 | 0.82 | 68 ± 19 | 46 ± 26 | 0.15 | 74 ± 25 | 75 ± 31 | 0.67 |
| **IgG, mg/dL**  Mean ± 1 SD | 1354 ± 725 | 1585 ± 658 | 0.40 | 1676 ± 1532 | 2006 ± 638 | 0.53 | 1758 ± 1989 | 1915 ± 1881 | 0.52 |
| **IgA, (mg/dL)**  Mean ± 1 SD | 162 ± 144 | 300 ± 185 | 0.03 | 616 ± 1270 | 430 ± 483 | 0.66 | 243 ± 705 | 232 ± 659 | 0.90 |
| **IgM, (mg/dL)**  Mean ± 1 SD | 344 ± 601 | 97 ± 62 | 0.20 | 63 ± 45 | 34 ±17 | 0.08 | 35 ± 34 | 32 ± 33 | 0.48 |
| **Kappa/Lambda ratio**  Mean ± 1 SD | 4.46 ± 7.97 | 1.68 ± 1.03 | 0.30 | 1.19 ± 1.37 | 17.45 ± 37.48 | 0.39 | 45.08 ± 135.07 | 67.38 ± 192.99 | 0.35 |
| **M-Spike, (g/dL)**  Mean ± 1 SD | 1.50 ± 0.96 | 0.68 ± 0.55 | 0.28 | 0.48 ± 0.28 | 1.66 ± 1.89 | 0.54 | 2.34 ± 2.22 | 3.41 ± 2.67 | 0.12 |
| **Beta-2 Microglobulin, (mg/L)**  Mean ± 1 SD | 6.5 ± 5.6 | 4.9 ± 3.9 | 0.49 | 2.48 ± .66 | 3.0 ± 1.98 | 0.77 | 4.1 ± 5.8 | 4.7 ± 5.8 | 0.45 |
| **Albumin, (g/dL)**  Mean ± 1 SD | 4.13 ± 0.42 | 4.01 ± 0.31 | 0.43 | 3.94 ± 0.45 | 3.90 ± 0.27 | 0.82 | 3.98 ± 0.49 | 3.81 ± 0.54 | 0.01 |

+: MGUS: monoclonal gammopathy of undetermined significance, SMM: smoldering multiple myeloma; MM: multiple myeloma.

++: P-values were computed using the Chi-square test for categorical variables and Welch’s Two Sample t-test for continuous variables.

*:High risk cytogenetics were the count of positive results for del17p, t(4:14), t(14:16), t(14:20), gain1q, and del1p.

^:Revised multiple myeloma International Staging System (R-ISS) was calculated using the International Myeloma Working Group formula with 166-245 (U/L) as the reference range for lactate dehydrogenase (LDH) and del17p, t4:14, and t14:16 as the high-risk cytogenetic abnormalities.

&:Estimated glomerular filtration rate (eGFR) was calculated from blood creatinine results using the Chronic Kidney Disease Epidemiology Collaboration (CKD-EPI) equation.
