# Supplementary material for: Genome-wide profiling of 5-hydroxymethylcytosines in circulating cell-free DNA reveals population-specific pathways in the development of multiple myeloma
Source: J Hematol Oncol. 2022 Aug 16;15:106. doi: 10.1186/s13045-022-01327-y (PMC9380317; doi:10.1186/s13045-022-01327-y)
Supplement: Supplementary file 2 — Additional file 2. Materials and Methods. [file 13045_2022_1327_MOESM2_ESM.docx]

**Materials and Methods**

**Study subjects.** We prospectively and systematically enrolled adult patients ≥20 years old with newly diagnosed MM and its precursors (i.e., MGUS and SMM) at the University of Chicago Medical Center since June, 2010. Informed consent was obtained from all participants. Blood samples and epidemiology questionnaire data were collected at the time of diagnosis. A total of 377 plasma (MM=324, SMM=20, MGUS=33) from a subset of patients diagnosed during 2010-2017 were obtained for cfDNA extraction and 5hmC-Seal profiling in the current report. Of the 377 plasma samples, 342 (MM=294, SMM=18, MGUS=30) passed quality control and were annotated with self-reported race/ethnicity as either EA (European American) or AA (African American). Clinical data were extracted from the electronic medical records. Fluorescent *in situ* hybridization (FISH) analysis of CD138^+^ plasma cells enriched from bone marrow aspirate samples with a panel of probes designed to target MM-associated cytogenetic abnormalities was performed at the University of Chicago Cancer Cytogenetic Laboratory. This study was approved by the University of Chicago Institutional Review Board (Approval No. 10-178B and 14-0482).

**Sample preparation, 5hmC-Seal profiling, and sequencing.** Detailed protocols for constructing the 5hmC-Seal libraries in cfDNA samples have been previously described.^1,2^ Briefly, circulating cfDNA was extracted from ~1-3 mL of plasma using the QIAamp Circulating Nucleic Acid Kit (Qiagen, Venlo, Netherlands) according to manufacturer’s protocols. DNA concentration was quantified using a Qubit fluorometer (Thermo Fisher Scientific, Massachusetts, USA). For each sample, ~2-4 ng of cfDNA was used to prepare the 5hmC-Seal libraries. The cfDNA samples were randomized for constructing 5hmC-Seal libraries and subsequent sequencing. First, the Illumina-compatible adaptors were ligated onto the cfDNA samples per our published protocols.^2^ Next, T4 bacteriophage β-glucosyltransferase was used to transfer an engineered glucose moiety containing an azide group onto the hydroxyl group of 5hmC-modified fragments. The azide group was then reacted with a biotin-conjugated strained alkyne DBCO-PEG_4_-biotin (Sigma, Missouri, USA) via click chemistry to biotinylated the DNA fragments. Streptavidin beads (Thermo Fisher Scientific, Massachusetts, USA) were used for affinity enrichment of 5hmC-containing DNA fragments. The 5hmC-Seal libraries were subsequently constructed through PCR amplification using KAPA HyperPlus Library Construction Kit (Roche, Basel, Switzerland) and paired-end sequenced (PE60) using the Illumina NextSeq500 platform (Illumina Inc., California, USA) at The University of Chicago Genomics Facility. Technical robustness of the 5hmC-Seal approach in cfDNA has been demonstrated in previous studies.^2,3^

**Bioinformatic processing.** We removed adapter sequences from raw sequencing reads using Trimmomatic.^4^ Low quality bases at the 5' and 3' ends were trimmed based on phred score to a minimum length of 30 bp. The sequencing reads were aligned to the reference genome hg19 using Bowtie2 with the end-to-end alignment mode.^5^ Read pairs were concordantly aligned with fragment lengths ≤ 500 bp, having, on average, no more than one ambiguous base and no more than four mismatched bases per 100 bp length. Alignments with Mapping Quality Score ≥ 10 were counted for overlap with the GENCODE^6^ gene bodies using featureCounts,^7^ without strand information. We excluded gene bodies with less than 10 fragment counts in more than 5% samples for further analysis. The raw fragment counts were then normalized using DESeq2,^8^ which performed an internal normalization that corrected for library size. Additionally, we obtained the histone modification data, particularly enhancer markers: H3K4me1 and H3K27ac, derived from various tissues, from the Roadmap Epigenomics Project.^9^ We summarized 5hmC-Seal read counts for these genomic features for comparison.

**Differential analysis between MM and its precursors.** To identify population-specific epigenetic mechanisms that may underlie the development of MM, we compared 5hmC features between MM and its precursors (MGUS+SMM, i.e., MGUS and SMM combined) in all patients. Genes with differentially modified gene bodies were identified using logistic regression controlling age, sex, and self-reported race/ethnicity. Comparing our identified differentially modified genes with published hydroxymethylome^10^ and gene expression data^11^ of MM cells supports MM relevance of our findings in cfDNA and suggests that some genes may play a role in both MM development and prognosis. Next, we evaluated to what extent the differential gene bodies differed between EA and AA patients. A Benjamini-Hochberg adjusted FDR^12^ of 5% was used to identify modified features that were statistically significantly different between EA and AA patients with MM.

In addition, we also compared gene bodies regarding 5hmC modification levels between MM and its precursors in EA and AA patients, separately. Genes with differentially modified gene bodies were identified using logistic regression controlling age and sex. Because the sample size was larger for EA, we used the number of top differential genes, instead of strict cutoff of false discovery rate (FDR), as a criterion for comparison between EA and AA. Using randomly sampling, we generated the null distribution of shared differential gene bodies between EA and AA patients. Specifically, for the top n (ranging from 1 to 2000) differential genes, n genes were randomly selected from the differential gene list obtained from EA and AA patients, separately, and counted for the number of shared genes. This process was repeated for 10,000 times for each n-gene set, and the mean number of shared genes was then calculated to construct the null distribution. All statistical analyses were performed using the R Statistical Computing Environment (v3.5.1).^13^

**Exploration of functional insights.** We used *clusterProfiler*^14^ to explore potential functions of identified genes with respect to Kyoto Encyclopedia of Genes and Genomes (KEGG) pathway.^15^ For each comparison, the top 500 differential genes were included in the analysis. Pathways with at least three gene counts and an FDR of 20% were considered significant. To understand the systems-level functionality of the differentially modified genes, we used the *CEMiTool* to reveal the modular gene co-expression network.^16^ To be more inclusive, top 500 differential genes was used for the co-expression network analysis. In addition, we used the protein-protein interaction from the Human Reference Interactome (HuRI)^17^ map to identify potential hub genes for the differentially modified 5hmC gene bodies. For each identified module, the Normalized Enrichment Score (NES) was calculated and shown in the figure. Red represents higher activity and blue represents lower activity. Specifically, the Gene Set Enrichment Analysis (GSEA) was performed using the component genes within a module as a gene set and the median z-score values of each population (e.g., EA and AA) as the rank.^18^ For each NES, a Benjamini-Hochberg adjusted p-value was calculated, and an adjusted p-value <0.05 was considered significant.^12^

**Supplementary References**

1. Song CX, Szulwach KE, Fu Y, Dai Q, Yi C, Li X, et al. Selective chemical labeling reveals the genome-wide distribution of 5-hydroxymethylcytosine. Nat Biotechnol 2011;29:68-72.

2. Li W, Zhang X, Lu X, You L, Song Y, Luo Z, et al. 5-Hydroxymethylcytosine signatures in circulating cell-free DNA as diagnostic biomarkers for human cancers. Cell Res 2017;27:1243-57.

3. Han D, Lu X, Shih AH, Nie J, You Q, Xu MM, et al. A highly sensitive and robust method for genome-wide 5hmC profiling of rare cell populations. Mol Cell 2016;63:711-9.

4. Bolger AM, Lohse M, Usadel B. Trimmomatic: a flexible trimmer for Illumina sequence data. Bioinformatics 2014;30:2114-20.

5. Langmead B, Salzberg SL. Fast gapped-read alignment with Bowtie 2. Nat Methods 2012;9:357-9.

6. Harrow J, Frankish A, Gonzalez JM, Tapanari E, Diekhans M, Kokocinski F, et al. GENCODE: the reference human genome annotation for The ENCODE Project. Genome Res 2012;22:1760-74.

7. Liao Y, Smyth GK, Shi W. featureCounts: an efficient general purpose program for assigning sequence reads to genomic features. Bioinformatics 2014;30:923-30

8. Love MI, Huber W, Anders S. Moderated estimation of fold change and dispersion for RNA-seq data with DESeq2. Genome Biol 2014;15:550.

9. Roadmap Epigenomics Consortium, Kundaje A, Meuleman W, Ernst J, Bilenky M, Yen A, et al. Integrative analysis of 111 reference human epigenomes. Nature 2015;518:317-30.

10. Chatonnet F, Pignarre A, Serandour AA, Caron G, Avner S, Robert N, et al. The hydroxymethylome of multiple myeloma identifies FAM72D as a 1q21 marker linked to proliferation. Haematologica 2020;105:774-83.

11. Zhan F, Huang Y, Colla S, Stewart JP, Hanamura I, Gupta S, et al. The molecular classification of multiple myeloma. Blood 2006;108:2020-8.

12. Benjamini Y, Hochberg Y. Controlling the false discovery rate: a practical and powerful approach to multiple testing. J R Stat Sco Ser B 1995;57:289-300

13. R: A Language and Environment for Statistical Computing. R Foundation for Statistical Computing, Vienna. 2013 at <http://www.R-project.org/> )

14. Yu G, Wang LG, Han Y, He QY. clusterProfiler: an R package for comparing biological themes among gene clusters. OMICS 2012;16:284-7.

15. Kanehisa M, Sato Y, Kawashima M, Furumichi M, Tanabe M. KEGG as a reference resource for gene and protein annotation. Nucleic Acids Res 2016;44:D457-62.

16. Russo PST, Ferreira GR, Cardozo LE, Burger MC, Arias-Carrasco R, Maruyama SR, et al. CEMiTool: a Bioconductor package for performing comprehensive modular co-expression analyses. BMC Bioinformatics 2018;19:56.

17. Luck K, Kim DK, Lambourne L, Spirohn K, Begg BE, Bian W, et al. A reference map of the human binary protein interactome. Nature 2020;580:402-8.

18. Subramanian A, Tamayo P, Mootha VK, Mukherjee S, Ebert BL, Gillette MA, et al. Gene set enrichment analysis: a knowledge-based approach for interpreting genome-wide expression profiles. Proc Natl Acad Sci U S A 2005;102:15545-50.
