# Supplementary material for: Genome-wide profiling of 5-hydroxymethylcytosines in circulating cell-free DNA reveals population-specific pathways in the development of multiple myeloma
Source: J Hematol Oncol. 2022 Aug 16;15:106. doi: 10.1186/s13045-022-01327-y (PMC9380317; doi:10.1186/s13045-022-01327-y)
Supplement: Supplementary file 4 — Additional file 4: Fig. S1. Supplementary results for the differential analysis between AA and EA patients with MM. Differential analysis was performed between AA and EA patients with MM for each gene body (5hmC modification levels, i.e., the normalized 5hmC-Seal read counts), using multivariable logistic regression models, controlling for age and sex. The heat map shows the 259 differential gene bodies at 5% FDR between AA and EA patients with MM. [file 13045_2022_1327_MOESM4_ESM.docx]

**Supplementary Figures**

**
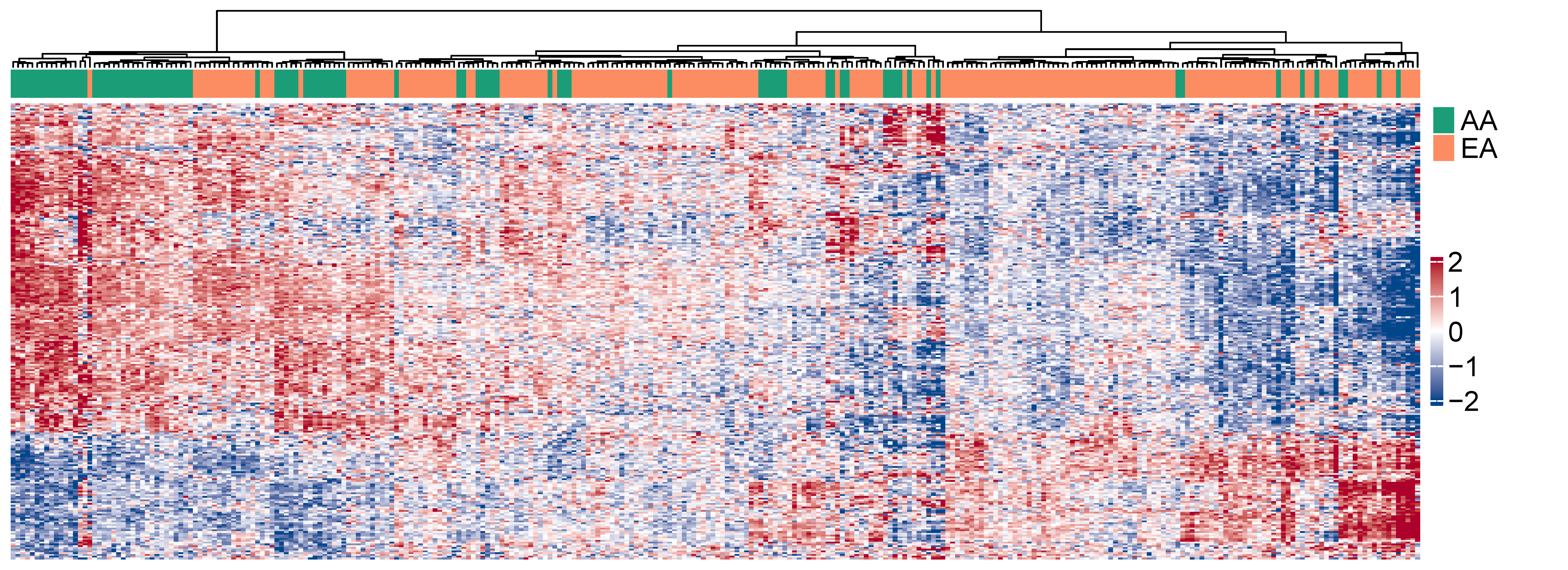
**

**Fig. S1. Supplementary results for the differential analysis between AA and EA patients with MM.**  Differential analysis was performed between AA and EA patients with MM for each gene body (5hmC modification levels, i.e., the normalized 5hmC-Seal read counts), using multivariable logistic regression models, controlling for age and sex. The heat map shows the 259 differential gene bodies at 5% FDR between AA and EA patients with MM.
